# Mean ± Standard Deviation Intake Values for 1–<10-Year-Old South African Children for Application in the Assessment of the Inflammatory Potential of Their Diets Using the DII^®^ Method: Developmental Research

**DOI:** 10.3390/nu14010011

**Published:** 2021-12-21

**Authors:** Sonia Malczyk, Nelia P. Steyn, Johanna H. Nel, Gabriel Eksteen, Linda Drummond, Wilna Oldewage-Theron, Mieke Faber, Martha E. van Stuijvenberg, Marjanne Senekal

**Affiliations:** 1 Department of Human Biology, University of Cape Town, Cape Town 7701, South Africa; soniamalczyk@gmail.com (S.M.); nelia.steyn@uct.ac.za (N.P.S.); linda@linda-drummond.com (L.D.); 2 Department of Logistics, Stellenbosch University, Stellenbosch 7602, South Africa; jhnel@sun.ac.za; 3 Department of Clinical and Experimental Endocrinology, KU Leuven University, 3000 Leuven, Belgium; gabrieljohannes.eksteen@kuleuven.be; 4 Department of Nutritional Sciences, Texas Tech University, Lubbock, TX 79409, USA; Wilna.Oldewage@ttu.edu; 5 Department of Sustainable Food Systems and Development, University of the Free State, Bloemfontein 9301, South Africa; 6 Non-Communicable Diseases Research Unit, South African Medical Research Council, Cape Town 7505, South Africa; mieke.faber@mrc.ac.za (M.F.); lize.vanblerk@gmail.com (M.E.v.S.)

**Keywords:** dietary inflammatory index, children 1–<10-years-old, mean intake, NCDs, South Africa, chronic low-grade inflammation

## Abstract

This study aimed to develop a set of mean ± standard deviation (SD) intake values for South African (SA) children for 36 of the 45 food parameters included in the original Dietary Inflammatory Index (DII^®^) tool. The SA food composition database contains 30 of the 45 food parameters included in the original DII^®^, and a supplementary database was developed for six of the food parameters not included in the SA database. The SA child mean ± SD intake of macronutrients, micronutrients and select flavonoids was calculated by age in years, using eight data sets from dietary surveys conducted in SA in the last three decades. A total sample of 5412 children was included in the calculation of the mean ± SD. The current study sample was determined to be representative of 1–<10-year-old children in SA, and the plausibility of the mean intake values was confirmed by being in line with age-appropriate recommendations. Furthermore, an increase in energy, macronutrient, and most micronutrient intakes with increase in age was evident. The generated mean ± SD values for SA children can be used for calculation of the inflammatory potential of the dietary intake of SA children in the age range of 1–<10-year-old children.

## 1. Introduction

Chronic inflammatory diseases are the most significant causes of death in the world [1,2]. As an intermediary risk factor for non-communicable diseases (NCDs), chronic low-grade inflammation (CLGI) is particularly relevant in a world where the incidence of NCDs is steadily increasing. It is now widely recognized that obesity, type 2 diabetes, cardiovascular disease, cancers, respiratory and auto-immune disorders, as well as arthritis and depression are associated with CLGI [3]. While many of these NCDs only present later in life, individuals can be exposed to NCD risk factors throughout the course of their life—even the maternal inflammatory environment has been linked to programming the fetus for potential chronic disease presentation later in life [4]. This warrants the examination of inflammatory risk factors in infants, children and adolescents, as prevention can play a large role in the battle against NCDs.

Arguably, one of the most important risk factors of CLGI is dietary intake. It has been suggested that unhealthy diets pose the largest risk to morbidity and mortality in general—greater than unsafe sex, and alcohol, drug, and tobacco use combined [5]. The dietary treatment of conditions such as metabolic syndrome has been shown to have a profound impact on the regulation of blood pressure, the control of plasma lipid levels, and the improvement of insulin resistance [6]. The impact of dietary intake on CLGI is similar. Dietary patterns (both pro- and anti-inflammatory), as well as several specific nutrients and other biologically active compounds in foods and spices all have a role to play in the inflammatory process. Dietary inclusion or exclusion of certain food parameters can thus have a marked impact on overall health. Due to the growing prevalence of dietary-related NCD risk factors, including raised blood pressure, overweight and obesity, as well as diabetes [7] in South Africa, the need for tailored tools to assess the inflammatory potential of the diets of South Africans is necessary.

Created as a tool for quantifying the dietary inflammatory potential of an individual’s diet, the Dietary Inflammatory Index (DII^®^) was developed by Shivappa et al. [8] to assess the health effects of diet-associated inflammation. The tool can be used to classify an individual’s dietary intake on a continual scale ranging from maximally anti-inflammatory to maximally pro-inflammatory [9]. The first version of the tool (Gen1) was published in 2009 [9] and a refinement thereof (Gen2) was published in 2014 [8]. Calculation of an individual DII-score is dependent on the availability of three sets of values, namely:

(1) A food parameter-specific inflammatory effect score, which can be pro- or anti-inflammatory [8], for 45 food parameters using findings on food parameter-inflammation associations from 1943 research papers (‘food parameters’ consist of whole foods, nutrients and other bioactive compounds);
(2) A composite database of mean and standard deviation (mean ± SD) intake of each of the 45 food parameters representing a wide range of diets across diverse populations, which for the DII^®^ was derived from data sets for adults mostly from 11 countries, the majority of which would not be considered low-to-middle-income countries (LMICs) such South Africa is; and
(3) Individual dietary data collected as part of a cross-sectional survey or intervention baseline/follow-up.

To calculate the individual DII score, the individual dietary data is firstly multiplied with the global mean ± SD to express each individual’s exposure relative to the ‘standard global mean’ as a Z-score. The Z-score is then converted to a percentile score to minimize the effect of ‘right skewing’. Shivappa et al. [8] explain that “the centered percentile value for each food parameter is then multiplied by its respective food parameter-specific inflammatory effect score to obtain the ‘food parameter specific DII score’. As a final step, all of the ‘food parameter-specific DII scores’ are summed to create the ‘overall DII score’ for an individual.”

However, Shivappa et al.’s [8] global mean ± SD adult intake composite data for the 45 parameters on the DII^®^ may not consider the range of dietary intake patterns in South Africa’s diverse populations. These include traditional diet patterns high in beans and legumes, vegetables, traditional meats, and maize meal porridge (pap), as well as “Western” patterns characterized by energy-dense, processed, high sugar/fat foods (white bread, processed and red meat, roast potatoes and chips, sweets (candy) and chocolate, soft drinks, and cheese) [10]. Moreover, the fortification of two of South Africa’s staple foods (bread and maize flour) with eight micronutrients (iron, zinc, vitamin A, thiamine, riboflavin, niacin, vitamin B6 and folate) [11] would not be reflected in the DII^®^ global database.

Khan et al. (2018) [12] developed a Children’s Dietary Inflammatory Index (C-DII) for 6–14-year-old children where the same food parameter-specific inflammatory effect scores for the food parameters were used, but the adult global mean ± SD database was replaced with a database generated for 6–14-year-old children for 25 of the 45 food parameters of the adult DII^®^ [12]. They used data from 16 countries, of which the majority were high- income countries. Dietary data from one small South African study (*n* = 157) were included [12,13]. Of note is that South Africa has the lowest 2021 Human Development Index (0.705) [14] of the 16 counties included in the development of the C-DII, which does raise the possibility that these values may not be appropriate for use in the South African setting. Moreover, Khan et al. [12] themselves expressed concern about the robustness of their child global mean ± SD values: “[there is] a relative paucity of robust and reliable data on child nutrition. Regular public health surveillance of child nutrition occurs infrequently around the world, and few research studies have collected children’s dietary data, so to find reliable sources for these data was challenging, and not all datasets provided 100% complete dietary data”. A further consideration is that the C-DII does not include the six classes of flavonoids [12] (flavan-3-ols, flavones, flavonols, flavonones, anthocyanidins and isoflavones) listed as food parameters in the DII^®^. These flavonoids all have strong anti-inflammatory effect scores [8]. Omission of these food parameters may thus result in false high C-DII scores that would reflect more pro-inflammatory diets.

Investigation of the inflammatory potential of the diets of South African children and those in other LMICs could provide significant insights for application in the development of dietary behavior change interventions for children. We therefore aimed to develop a set of South African child mean ± SD values for parameters on the DII^®^ [8] using dietary intake data from South African children aged 1-to-younger than 10 years old. Not only would these values reflect the cultural diversity of dietary patterns and fortification of staple foods in South Africa, but we also aimed to develop a food flavonoid content database to generate South African child mean ± SD values for these important anti-inflammatory dietary compounds.

## 2. Materials and Methods

### 2.1. Identification of Quantified Dietary Surveys (South African Children)

A comprehensive review of the literature was conducted to identify published studies on dietary intake in South African children. This review expanded on the findings of Steyn et al. [15] and employed similar methods. It involved both electronic and manual searching of peer reviewed and grey literature, as well as electronic data sets of national and local studies done on the dietary intakes of South African children aged 1–10-years since 1990. Two main platforms were used as part of the search strategy: MEDLINE and EBSCOHost. Both platforms facilitated a filtered search for studies conducted in South Africa. MEDLINE is a peer-reviewed bibliographical database of life sciences and biomedical information and the largest subset of PubMed. This search engine allowed us to conduct an “advanced search” with our targeted key words. EBSCOHost facilitated the review of five separate databases (Academic Search Premier, Africa Wide studies, CINAHL, Health Source (Nursing Education), and PsychINFO). This expanded the scope of our comprehensive literature review, as it included databases not directly aimed at nutrition research. Databases were searched for publications which included some or all of the following key words in either the title, abstract or key words: “dietary intake”, “food intake”, “nutrient intake”, “caloric intake”, “energy intake”, “food habits”, “dietary trends”, “diet survey(s)”, “dietary survey(s)”, “nutrition survey(s)”, “food consumption”, “dietary assessment(s)”, “nutrition assessment(s)”, “nutritional assessment(s)”, “nutritional status”, and “micronutrient intake”. The search was also limited to studies that contained the words “child”, “children”, “infant”, “baby”, “babies”, “toddler(s)”, and either “South Africa” or “South African”.

Studies that met the following criteria were included: (1) one of the following dietary intake methods used for data collection: 24 h recall, quantified food frequency questionnaire (FFQ), weighed or estimated dietary record, dietary history to record children’s nutrient intakes, and/or foods consumed; (2) participants 1–<10-years old (both pre-school and primary school-aged children); and (3) food intake data available as total gram per day with an allocated food code. Studies that focused specifically on the dietary intake of children with a particular disease condition, e.g., diabetes or human immunodeficiency virus/acquired immunodeficiency syndrome (HIV/AIDS) were excluded.

**Figure 1:**
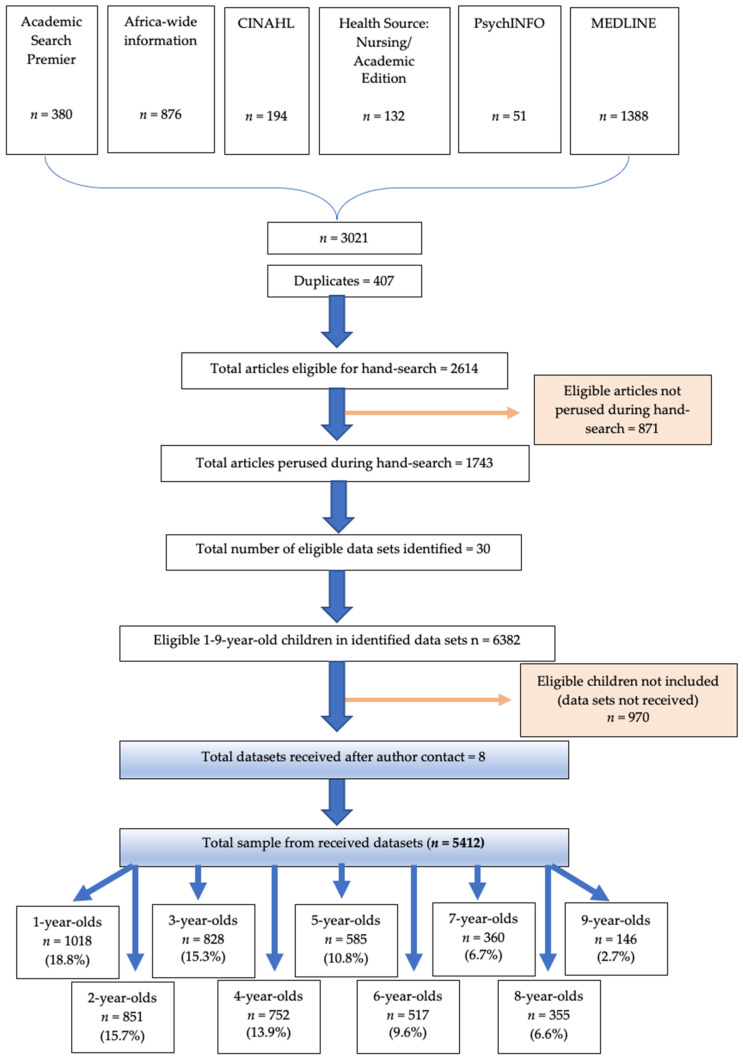
Flow diagram of search strategies and results.

Figure 1 presents a flow diagram of the search strategy and results of the comprehensive literature review. The literature search identified 2551 articles eligible for the hand-search for the identification of South African studies which included dietary intake data within the correct age range (1–<10-years-old) (Figure 1). The hand-search was concluded when a total of 30 eligible studies with a total of 6382 eligible children had been identified, at which point a total of 1281 articles/studies of the 2551 had been perused. The primary investigators (PIs) of these 30 studies were contacted with a request for use of their dietary intake data. We received eight datasets, which included a total of 5412 participants and their respective food intake parameter codes and amount consumed in grams. This research was secondary data analysis. All data was obtained from the primary researchers who had consent and ethical approval in place. A total of 970 eligible 1–<10-year-old children were not included because data sets could not be secured from PIs. The age range of the final sample was as follows: 1-year-olds *n* = 1018 (18.8% of total sample), 2-year-olds *n* = 851 (15.7%), 3-year-olds *n* = 828 (15.3%), 4-year-olds *n* = 752 (13.9%), 5-year-olds *n* = 585 (10.8%), 6-year-olds *n* = 517 (9.6%), 7-year-olds *n* = 360 (6.7%), 8-year-olds *n* = 355 (6.6%) and 9-year-olds *n* = 146 (2.7%) (Figure 1 and Table 1).

Dietary assessment methodologies varied between data sets (Table 1). While all studies employed a 24 h recall, only three studies repeated the recall in a subsample [13,16,17], and only one study [18] adjusted the single day’s intake to reflect usual intake using two additional recalls obtained in a sub-sample. The person interviewed in each study also varied. The majority of studies interviewed the mother or primary caregiver of the children [17,19,20,21], three studies interviewed the child in the presence of the mother/primary caregiver [16,18] and one study interviewed the children themselves [13]. All studies made use of the version of the South African Medical Research Council Food Composition Tables and/or the version of FoodFinder (version: 1.0, https://foodfinder.samrc.ac.za/, accessed on 28 January 2021) that was available at the time of study completion.

### 2.2. Generation of a Nutrient Databases for Food Parameters in the DII^®^

As mentioned, the C-DII [12] global child database only included mean ± SD intake values for 25 out of the 45 food parameters included on the adult DII^®^ [8]. To address this limitation, we aimed to generate a mean ± SD intake value for the majority of the 45 food parameters using raw data obtained from dietary intake studies conducted in South African children, described above (Table 1). However, the South African Medical Research Council (SAMRC) Composition database [31] currently only includes 30 of the 45 food parameters of interest. To address this constraint, a supplementary nutrient composition database was built that included values for six additional food parameters included in the original list of 45 (Table 2) for all food items/codes (*n* = 1758) that were consumed by participants in the eight obtained data sets. The following publicly available databases were used for these purposes: the United States Department of Agriculture (USDA) Database for the Flavonoid Content of Selected Foods (for flavan-3-ols, flavones, flavonols, flavanones and anthocyanidins content) [32], and the USDA Database for the Isoflavone Content of Selected Foods (for isoflavone content) [33]. In the event that a particular coded food item used in one of the eight South African datasets could not be found in either of the two USDA databases, the online database Phenol-Explorer.eu [34] was used as a final search reference.

All food codes included in the eight South African datasets were added to the supplementary database, organized chronologically, and then filtered for duplicates. All codes were then scanned and assigned a category: the codes that included multiple ingredients (e.g., recipes for soups, stews, casseroles, etc.) were broken down into their composite codes and proportions. Next, all codes and their respective breakdown proportions were labelled as either P (plant-based), A (animal-based) or M (mixed). As the polyphenol content of animal-based products is zero [35], it could be assumed that the polyphenol values for these codes were zero. The description of all plant-based codes and all mixed codes were searched for in the aforementioned USDA databases, to determine the mass per 100 g of each food parameter of flavan-3-ols, flavones, flavonols, flavanones, isoflavones and anthocyanidins. If none of these sources had listed values for these nutrients, cells were left blank (and for the purposes of the mean ± SD intake value calculation, assumed to have zero content of these nutrients). Records were kept of any USDA codes that were used as alternatives for any food codes for which there was no direct match (e.g., “Coriander leaf, dried” used for “Coriander leaf, fresh” as the closest match).

The polyphenol content for all codes that had an associated recipe breakdown was calculated by multiplying the polyphenol content of each recipe component (i.e., code) by the proportion it represented in the respective recipe. For example, code 3711 for “Salad: Carrot, Raw” has a recipe breakdown as follows: 70% code 3709: “Carrot, Raw (flesh and skin)”, 20% code 3532: “Apple, Average, Raw” and 10% code 3561: “Orange, Juice, Fresh”. The total flavan-3-ol, flavone, flavonol, flavanone, anthocyanidin and isoflavone content was first calculated for each of the individual codes (3709, 3532 and 3561), and then multiplied by its corresponding recipe proportion (0.7, 0.2 and 0.1) to determine the overall polyphenol content for code 3711.

### 2.3. Calculation of the South African Child Mean ± SD

As mentioned, eight original food intake data sets were received from the PIs of SA dietary studies and surveys who were willing to collaborate on the development of the mean ± SD intake values for SA children. The dietary intake methodology of each of the data sets is summarized in Table 1. These data sets included the details of participant’s age and gender, as well as original food codes and amounts (grams) consumed. All data sets were first reviewed, cleaned, and food codes checked and sorted as described in the previous section. Any data sets that included “old” food codes were re-coded to use the most recent or “new” food codes. The data of participants who fell above or below our designated age range (1–<10-years-old) were removed from the data sets.

### 2.4. Statistical Analyses

All raw data was combined into one data set to represent SA children, and the mean ± SD intake of each of the 36 food parameters were calculated. Calculations were done for each of the nine year-age groups. In larger studies, ages are typically grouped; therefore, calculations were also done for the three Provincial Dietary Intake Study (PDIS) age groups (age group 1 = 1–<3 years, age group 2 = 3–<6 years, age group 3 = 6–<10 years) [23].

To test the assumption that mean ± SD values would increase with age, based on the Dietary Reference Intakes (DRIs) for children from 1–<10-years old, these values were compared between the individual year groups and the three PDIS age groups using the Bonferroni multiple comparison test. Mean ± SD values were also compared descriptively with available DRI values to provide context to alignment of the intake found for the SA-sample with recommendations. The purpose of this comparison with DRIs was not to comment on the adequacy of the intake of this sample, but rather provide perspectives on the plausibility of the mean ± SD results for the various age groups.

## 3. Results

Table 3, Table 4, Table 5 and Table 6 present the mean ± SD by individual year of age calculated for each of the 36 food parameters, as compared with their respective DRI values where available. Several general trends were noted between individual years of age. Data revealed a general increase in energy, macronutrient, and most micronutrient intakes with increased years of age, with the exception of the dietary intake trends for calcium, vitamin C and vitamin D. The highest intake of calcium was seen in the 1-year-olds, followed by the 7-year-olds, and then the 2-year-olds; calcium intake for the other ages (3-, 4-, 5-, 6-, 8- and 9-year-olds) was significantly lower. Vitamin C intake was highest in the 7-year-olds, and then differed significantly amongst the other years of age. Vitamin D intake was significantly lower in the 4-year-olds than all other age groups. Flavan-3-ols intake increased significantly from 2-years-old and upwards, as did the added sugar intake.

Appendix ATable A1, Table A2, Table A3 and Table A4 present the means ± SD by the three age groups used in the 2018 PDIS: age group 1 comprised of 1–2-year-olds *n* = 1869 (34.5% of total sample), age group 2 comprised of 3–5-year-olds *n* = 2165 (40%) and age group 3 comprised of 6–9-year-olds *n* = 1378 (25.5%). Data revealed a general increase in energy, macronutrient, and most micronutrient intakes with each age group, with the exception of saturated fat, cholesterol, added sugar, calcium, vitamin A, vitamin C, vitamin E, flavan-3-ols, flavones, and caffeine. Saturated fat, vitamin E, flavones and vitamin A intake only increased significantly from age group 2 to age group 3. Cholesterol, added sugar, flavan-3-ol and caffeine intake increased significantly from age group 2, and then no further significant increase was noted between age group 2 and age group 3 for these four food parameters. Calcium intake was significantly higher in age group 1, and Vitamin C intake was significantly lower in age group 2 than the other age groups.

## 4. Discussion

This research set out to develop mean ± SD values for 1–<10-year-old South African children for 36 food parameters included in the original DII^®^ [8] to apply in the calculation of the inflammatory potential of dietary intake of children in this age range. The validity of these mean ± SD intakes, thus to what extent the values represent the true intake of children in the target age range, is critiqued below in terms of the representativeness of the study sample of 1–<10-year-old children used to calculate the values, the plausibility of the values in terms of age appropriate recommendations, the validity and reliability of the dietary methodology applied by researchers in the eight studies from which data sets were obtained, and lastly, the standardization of the data obtained for calculation of the mean ± SD values.

### 4.1. The Representativeness of the Sample Used to Calculate the Mean ± SD Values of 1–<10-Year-Old South African Children

Eight out of thirty potentially eligible data sets were obtained, of which one was representative of all provinces in the country (1999), one was representative of Gauteng and the Western Cape (2018) and six were convenience samples, one from KwaZulu-Natal (KZN) (2016), one from the Free State (2010), one from the Northern Cape (2014), one from the Eastern Cape (2017), one from Gauteng (2012) and one which included participants from Limpopo, KZN, Western Cape and Northern Cape (2015). Based on the 30 identified studies (Figure 1), the total number of eligible 1–<10-year-old children in the potentially eligible data sets was 6382, of which 5412 (84.8%) were included in the calculation of the SA mean ± SD values. The composition of the sample underpins the representativeness of the mean ± SD values generated for South African children included in the 30 studies. Inclusion of the data from the other studies may have increased the total sample with approximately 970 additional children (for a total sample of 6382). However, it is worth noting that 72.8% of these additional children were between 1–5 years, an age range that is already adequately covered in the current data set (*n* = 4034). Furthermore, a total sample of 5412 children in the age range of 1–<10-years is larger than any national survey sample with quantified dietary intake data in this age group that has been conducted in South Africa in the last 20 years; most notably, the National Food Consumption Survey *n* = 2894 [19]. This further supports the representativeness of the current study sample.

### 4.2. The Plausibility of the Mean ± SD Intake Values: Alignment with Age-Appropriate Recommendations

The total energy intake of the study sample increased significantly from the youngest (1-year-olds) to the oldest age group (9-year-olds) as could be expected based on the estimated energy requirement (EER) calculated using an active physical activity level (PAL) [36] and research by others [2,37,38,39,40,41]. In line with increases in total energy intake, total carbohydrate, fat, and protein intakes also increased with age, with significant differences between 1- and <10-year-olds. Similar to increases in energy intake, the increases in macronutrient intake with age are also in line with the findings of other researchers [2,37,38,39,40,41]. While DRIs are not available for total fat intake, it is evident that carbohydrate intake for all ages was above the EAR, but within a range that has been reported by others for carbohydrate intake in similar age groups [2,37,42]. Total protein intake also increased significantly with age, which is in agreement with the protein intake reported for South African [41] and rural Zambian [2] children of a similar age range. Dietary fiber intake also increased with age, with the intake of 1-year-olds being significantly lower than that of 9-year-olds. Dietary fiber intake was consistently below the EAR in all year groups. South African children have been shown to not meet the fruit and vegetable intake recommendations, be more likely to eat white rather than brown bread, to eat energy-dense nutrient-poor foods or snacks, i.e., salty snacks (crisps), sweet snacks (sweets, chocolate, cake, biscuits) and to drink sugar-sweetened beverages (SSBs), contributing to energy, carbohydrate and fat intake, without providing fiber [18,19,43,44,45]. Added sugar and trans-fat intakes also increased with age, but the increase was notably more prominent between the 1- and 2-year-olds, reflecting the possible increased exposure to energy-dense foods and drinks from two years of age onwards. This is in line with the high consumption prevalence of commercially produced foods and beverages during the complementary feeding period in LMICs [46].

Mineral intake had a general upward trend aligned with an increase in energy intake and age, with the exception of calcium which was consistently well below the EAR for all age groups. Intake of the other minerals is in line with the respective EARs and AIs, as well as the values of other national [47] and international studies [2,37,39], confirming the plausibility of results for mean ± SD intakes of minerals. In line with the current SA mean intakes reported herein, the intakes of phosphorus, magnesium, iron, zinc, and selenium in Greek children aged 1–13 years also all increased with age [39]. Trends for the South African child mean intake of the majority of vitamins also increased significantly with increasing age. This is in line with increasing vitamin intakes seen in pre-school children from the Free State in SA [41] as well as vitamin intakes described by Mitsopoulou et al. [39]. Vitamin D was the one exception in the current and other studies [39,41] where vitamin D intake was well below the EAR and did not show a strong trend of increasing with age. Usual vitamin intakes in Zambian children aged 4–8 years also generally increased with age with the exception of folate, which decreased from the 4–<5-year-olds to the 5–8-year-olds [2].

Research examining dietary flavonoid and isoflavone intake in children is unfortunately sparse, and it is therefore more difficult to compare the results of this study with other available data. While there are no fixed DRIs for these nutrients as yet, the general expectation was to note an increasing intake with increasing age and energy intake; however, this pattern was not as clear as it was with other micronutrients in this particular study. It also appears that other researchers were not always able to discern clear trends for dietary flavonoid intakes in children. The Dortmund Nutritional and Anthropometric Longitudinally Designed (DONALD) study in Germany estimated the intake of total flavonoids and isoflavones in 1312 children between 1985–2016 [48]. These researchers noted an increasing trend for total flavonoid intake with increasing age in both boys and girls aged 3–12 years; however, similar to the current study, they did not observe an obvious trend for total isoflavone intake with increasing age [48]. Total polyphenol intake by season (winter versus spring) was also explored in an Italian study involving 8–10-year-old children [40]. This study [40] noted lower median intakes of total flavonoids than German colleagues [48], as well as differing median intakes for flavan-3-ols, flavanones, flavones and isoflavones when compared to the current study. While intakes for flavonols and anthocyanidins were more comparable between the Italian [40] and the current study, the aforementioned differences emphasize the need to further develop and include these nutrients in national food composition databases. There is a lack of dietary polyphenol intake data in children worldwide; however, this was addressed in this South African study and thus highlights a major strength of the current study.

### 4.3. The Validity and Reliability of the Dietary Methodology Applied by Researchers

Dietary intake assessment in children is complex, with several key challenges including dietary intake of small amounts of food at frequent intervals, difficulties in estimating portion sizes [49], limited food recognition skills, memory constraints and limited concentration spans [50]. It has been suggested that children under the age of 12 years generally have difficulty reporting their dietary intake without parental assistance [50]. However, Livingstone et al. explained that from the age of 7–8-years, an unassisted recall can be considered if it covers intake over the immediate past, i.e., within the previous 24 h [51]. Other researchers confirm this, suggesting that children over the age of 8 years are likely to report their dietary intake even more accurately than their parents [50]. Some of the best methods described to overcome the dietary intake assessment challenges include a ‘consensus’ recall method, whereby the child (aged 8–10 years or above) and parent are interviewed together as this is suggested to improve the accuracy of recalls [50]. For younger children who may be in the care of a school or nursery for parts of the day, combining meal-time observations with parental reports may also help to improve accuracy [50]. A systematic review by Burrows and co-authors that examined the validity of dietary intake assessment methods in children, concluded that a 24 h multiple pass recall conducted over at least a 3-day period using parents as reporters, is the most accurate method of determining energy intake in 4–11-year-olds. They also found that weighed food records are most accurate for 0.5–4-year-olds [52]. Another systematic review examining the best dietary assessment methods for micronutrient intake in children concluded that the 3-day weighed dietary records was superior to the FFQ [49]. With all of this considered, it is pertinent to compare the different techniques employed by the various researchers who contributed their data to the current research study.

Of the data sets received for use in the calculation of the South African child mean ± SD intake, all except one [19] reported the use of a multiple pass interviewing method for the 24 h recalls employed in their research. Almost all authors commented on the validity of the 24 h recall as a dietary intake method. Several authors suggested that the validity of their 24 h recall was strengthened by the inclusion of both weekdays and weekend days in their total group of participants [16,17,18,20,22]. Faber and colleagues noted that a single 24 h recall allows for reporting of group means only and is likely not as good reflection of some nutrients (such as vitamin A) due to the large day-to-day as well as seasonal variation in some nutrient intakes [20]. Nel and colleagues commented on the disparity between vitamin A content reported by their 24 h recall versus a quantified liver intake frequency questionnaire, proposing that their 24 h recall overestimated the intake due to the exceptionally high vitamin A content of liver and suggested that the 24 h recall is not a suitable tool for assessing vitamin A intake in areas where liver is often consumed [21].

Other researchers, including van Stuijvenberg et al., suggested that the 24 h recall included in their research gave an accurate reflection of habitual intake due to their geographically isolated and low socioeconomic population with low dietary diversity [22]. Steyn and colleagues used a second and third 24 h recall from 146 participants and employed the US National Cancer Institute (NCI) adjustment method [24] to adjust dietary intake values to be a better reflection of true intake [18]. Some of the studies relied on the mother/primary caregiver alone, without input from schools or nurseries [21,22] or without input from the children themselves [19]. Finally, the ‘consensus’ recall method [50] was employed by three studies [16,23,53], which is encouraging.

Based on this critique, it is clear that there was some variety in the dietary assessment methodology employed by collaborators of the current study. While none of the researchers in any of the eight included data sets employed the “best practice” methods described by Burrows [53], the researchers’ various adjustment techniques were reasonable attempts to address the limitations of the 24 h recall dietary intake method. Thus, the nutrient means ± SD generated can be considered to be a reasonable estimation of the true intake of children aged 1–<10-years-old in South Africa.

### 4.4. The Standardization of the Data Obtained for Calculation of the Mean ± SD Values

The fourth and final inspection point of the validity and reliability of the SA child mean ± SD intakes presented in this study is the standardization of the data obtained for the calculation of these mean intakes. All data collected from other South African researchers included raw codes and gram amounts. This provided consistency in data from across the country due to the collective use of the SA food composition database. Furthermore, our methodology included re-analysis of this raw data to culminate in a completely updated dietary intake database. All nutrient values are therefore based on the most current version of the South African food composition database as well as a supplementary nutrient composition database created through this research, which included the addition of six polyphenols. This is considered a material strength of the study design, as no other researchers have done this.

### 4.5. Study Limitations

The results of this developmental research should also be considered within the context of several limitations. As indicated, not all the articles identified with the described search strategy were hand-searched. Several of the authors that were contacted with a request to use their data either did not respond to the enquiry or were not able to share their raw data sets in the format that was requested. Furthermore, it is important to consider that the spread of age by years in the total study sample for calculation of the SA child mean ± SD intake was not uniform, with the highest number of participants being 1-year-old (18.8%), with proportions consistently decreasing with age, with the lowest number of participants in the oldest age category of 9-years-old (2.7%). Finally, only approximately 44% of the nutritional information included in the most recent South African Food Composition database originates from South African data (FoodFinder, version: 1.0, https://foodfinder.samrc.ac.za/, accessed on 28 January 2021).

## 5. Conclusions

We successfully developed a set of plausible mean ± SD intake values for SA children for 36 food parameters in the DII^®^ [9] by applying a robust process, i.e., using standardized dietary intake data of a representative sample of 1–<10-year-old South Africans derived from national and local dietary intake studies of which the dietary methodology was deemed to be of acceptable quality. The SA child mean ± SD intake values include an additional 11 food parameters when compared to the C-DII [12]. These additional food parameters include several strong anti-inflammatory compounds such as flavones, flavanols, isoflavones, flavan-3-ols, flavanones, anthocyanidins, *n*-3 fatty acids, *n*-6 fatty acids, caffeine, green/black tea, and onion, which is deemed a major strength of our outputs [8].

The SA child mean ± SD intake values can be used by researchers and healthcare practitioners to assess the inflammatory potential of any 1–<10-year-old SA child’s diet. Future research should consider extending the use of the tool to include older South African age groups, such as adolescents and adults by developing mean ± SD intake values representative of these age groups. The flavanol supplementary database we developed, that has food codes that are aligned with the SAMRC food composition database food codes, is available upon request. This supplementary database can be used by any researcher with South African dietary intake data that can link a SA MRC food code that was included in the supplementary database to their food item, making a large contribution to the assessment of the inflammatory potential of the diet of South African children.

## Figures and Tables

**Table 1:** Detail of dietary intake methodology for data sets used to calculate the South African childhood mean ± SD intake.

Name of Dataset	Data Set 1	Data Set 2	Data Set 3	Data Set 4	Data Set 5	Data Set 6	Data Set 7	Data Set 8	PDIS 2018	Totals
Source of Dataset	[19]	Oldewage-Theron (unpublished)	[13]	[16]	[21,22]	[17]	[17]	[20]	[23]	
Study Location	National	Gauteng	Free State	Eastern Cape	Northern Cape	KZN	KZN	Limpopo + KZN + Western Cape + Northern Cape	Western Cape + Gauteng	
Study sample	2858	15	141	236	150	205	205	592	1326	
Total	1–9 years	5–14 years	8–14 years	6–18 years	2–4 years	1–2 years	1–2 years	1.5–6 years	1–9 years	
Age Range	1440	6	70	112	77	102	101	300	655	
Male	1418	9	71	122	73	104	104	285	671	
Female										
Unknown	15	-	-	2	-	-	-	7	-	
1 year	450	0	0	0	1	195	194	22	156	1018 (18.8%)
2 years	443	0	0	0	54	10	10	157	177	851 (15.7%)
3 years	411	0	0	0	44	0	0	174	199	828 (15.3%)
4 years	386	0	0	0	51	0	0	154	161	752 (13.9%)
5 years	358	1	0	0	0	0	0	72	154	585 (10.8%)
6 years	338	0	0	18	0	0	0	8	153	517 (9.6%)
7 years	210	0	0	20	0	0	0	1	129	360 (6.7%)
8 years	215	0	1	17	0	0	0	0	122	355 (6.6%)
9 years	48	2	4	17	0	0	0	0	75	146 (2.7%)
								Total Sample: 1–9 years		5412
Excluded: unknown age OR out of age range	15	13	136	164	0	0	0	6	0	334
Dietary intake method	Single 24-h recall	Single 24-h recall (weekday)	3 repeated 24-h recalls, including 1 weekend day)	Repeated 24-h recalls (2 days-a weekday& weekend day)	Single 24-h recall	Two 24-h recalls (one week apart)	Two 24-h recalls (one week apart)	Single 24-h recall	Single 24-h recall	
Adjustment of single day to reflect usual intake	No	No	Not applicable	No	No	Not applicable	Not applicable	No	Yes-2nd recall: 148, 3rd recall: 146 Adjustment NCI method [24,25].	
Person interviewed	Mother/primary caregiver	5–14-year-olds: child in presence of mother/primary caregiver	Children themselves	6–8-year-olds: child in presence of mother/primary caregiver 9–18-year-olds: children themselves	Mother/primary caregiver	Mother/primary caregiver	Mother/primary caregiver	Mother/primary caregiver	1–5-year-olds: mother/primary caregiver 6–9-yearold’s: child in presence of mother/primary caregiver	
Interviewing process	Not described	Four-stage multiple pass interviewing method [26]	Four-stage multiple pass interviewing method [26]	Four-stage multiple pass interviewing method [26]	Multiple pass method	Multiple pass method	Multiple pass method	Multiple pass method	Multiple pass method [27]	
Interviewers	Field workers trained: video; field manual: guidance for questionnaires	Fieldworkers 3-day training: ethics, research philosophy and questionnaire administration	Fieldworkers 3-day training: ethics, research philosophy and questionnaire administration	Fieldworkers 2-day training: ethics, general research philosophy and questionnaire administration	Registered dietitian working in the area; trained by study supervisors	Full-time employed fieldworkers with at least 10 years of experience in collecting 24-h recall data	Full-time employed fieldworkers with at least 10 years of experience in collecting 24-h recall data	Locally recruited and trained field workers (grade 12+); week training session	Field workers week training: anthropometry, questionnaire administration; 24-h recall administration	
Portion size estimation details	Wax/foam models of HH utensils; dry foods; empty containers	Generic 3D food models [28] and locally used HH utensils	Generic 3D food models [28] and locally used HH utensils	Generic 3D food models [28] and locally used HH utensils	Food models and HH utensils	Household utensils, food containers and wrappers, plastic food models, three-dimensional food models	Household utensils, food containers and wrappers, plastic food models, three-dimensional food models	Household utensils, food containers and wrappers, plastic food models, three-dimensional food models	Life size sketches: HH utensils, crockery, sketches of foods, generic 3D flour food models [29]	
Database and software used	SA MRC Foodfinder program (1998 database)	SA MRC FoodFinder program	SA MRC FoodFinder program	SA MRC FoodFinder program	SA MRC Food Composition Tables [30].	SA MRC Food Composition Tables [30].	SA MRC Food Composition Tables [30].	SA MRC Food Composition Tables [30].	SA MRC Food Composition Tables: 2017 [31]. *	
Quality control	Registered dietitian; data entry and analyses completed under the supervision of 2 statisticians.	Registered dietitian	Registered dietitian	Registered nutritionist (SA)	Registered dietitian completed coding and entered data; data entry checked by the project leaders.	Each 24-h recall was coded by two independent fieldworkers; coded data checked by experienced researcher.	Each 24-h recall was coded by two independent fieldworkers; coded data checked by experienced researcher.	Data was coded by experienced dietitian and checked by researcher	Experienced dietitians (fieldwork managers) checked each questionnaire for quality and completeness and coded all recalls.	

EC = Eastern Cape; KZN = KwaZulu-Natal; NCI = National Cancer Institute; SAMRC = South African medical Research Council; HH = Household; E = Energy; FSM = Food Service Management; 24-h = 24 h. * FoodFinder 3 (2019 database) Total E and added sugar values were adjusted as necessary for changes following implementation of Health Promotion Levy in April of 2018; Sodium content of food items were adjusted as necessary for changes following implementation of the Sodium Reduction Regulations in 2013.

**Table 2:** Available, additional, and excluded food parameters used to develop the mean ± SD intake values for South African children, based on the 45 food parameters included in the original DII^®^.

DII^®^ Food Parameters Available in the South African Medical Research Council (SAMRC) Food Composition Database (*n* = 30)		DII^®^ Food Parameters Added to the SA Child Mean ± SD Intake Values Supplementary Database (*n* = 6)	DII^®^ Food Parameters Excluded from the SA Child Mean ± SD Intake Values Database (*n* = 9) *
Vitamin B_12_ (mg)	Saturated fat (g)	Flavan-3-ols (mg)	Alcohol
Vitamin B_6_ (mg)	Selenium (mg)	Flavones (mg)	Eugenol
Carbohydrate (g)	Thiamin (mg)	Flavonols (mg)	Garlic
Cholesterol (mg)	*Trans*-fat (g)	Flavanones (mg)	Ginger
Energy (kcal)	Vitamin A (RE ^3^)	Isoflavones (mg)	Saffron
Total fat (g)	Vitamin C (mg)	Anthocyanidins (mg)	Turmeric
Fibre (g)	Vitamin D (mg)		Pepper
Folic acid (mg)	Vitamin E (mg)		Thyme/Oregano
Iron (mg)	Zinc (mg)		Rosemary
Magnesium (mg)	β-carotene (mg)		
MUFA ^1^ (g)	*n*-3 Fatty acids (g)		
Niacin (mg)	*n*-6 Fatty acids (g)		
Protein (g)	Caffeine (mg)		
PUFA ^2^ (g)	Onion (g)		
Riboflavin (mg)	Green/black tea (g)		

^1^ MUFA = Monounsaturated Fatty Acid; ^2^ PUFA = Polyunsaturated Fatty Acid; ^3^ RE = Retinol Equivalent; SA = South African. * Alcohol not relevant for 1–<10-year-old children; no data for level of consumption of the remaining items is available.

**Table 3:** South African child mean ± SD intake of energy and macronutrients by year of age.

		1 Year	2 Years	3 Years	4 Years	5 Years	6 Years	7 Years	8 Years	9 Years
	*n* =	1018	851	828	752	585	517	360	355	146
Energy (kJ)* EER based on PAL = active	EER * Boys	4393	4393	6213	6552	6937	7289	7699	8079	8548
	Girls	4166	4166	5837	6171	6514	6870	7192	7573	7908
	SA Mean	4447.0	4854.7	5138.2	5327.8	5830.4	6079.5	6490.6	6412.0	7110.4
	(±SD)	(1998.7)	(2084.3)	(2196.1)	(2112.5)	(2520.3)	(2402.7)	(2629.6)	(2617.0)	(2854.8)
	Bonferroni	(E)	(DE)	(D)	(D)	(C)	(BC)	(B)	(B)	(A)
Energy (kcal)	EER Boys	1046	1046	1465	1546	1638	1722	1820	1911	2018
	Girls	992	992	1375	1455	1537	1622	1699	1790	1865
	SA Mean	1063.9	1161.4	1229.2	1274.6	1394.8	1454.4	1552.8	1534.0	1701.0
	(±SD)	(478.2)	(498.6)	(525.4)	(505.4)	(603.0)	(574.8)	(629.1)	(626.1)	(683.0)
	Bonferroni	(E)	(DE)	(D)	(D)	(C)	(BC)	(B)	(B)	(A)
Total Protein (g)	EAR	-	-	-	-	-	-	-	-	-
	SA Mean	29.5	35.7	37.5	39.1	42.5	43.3	47.1	47.5	51.0
	(±SD)	(17.4)	(19.1)	(18.2)	(18.8)	(21.6)	(19.7)	(22.7)	(23.4)	(23.0)
	Bonferroni	(E)	(D)	(D)	(CD)	(C)	(BC)	(AB)	(AB)	(A)
Plant Protein (g)	DRI	-	-	-	-	-	-	-	-	-
	SA Mean	14.6	16.5	18.2	19.4	21.7	23.5	24.4	24.0	25.0
	(±SD)	(9.1)	(8.8)	(10.0)	(10.1)	(11.9)	(11.5)	(12.9)	(13.1)	(11.2)
	Bonferroni	(F)	(EF)	(DE)	(CD)	(BC)	(AB)	(A)	(A)	(A)
Animal Protein (g)	DRI	-	-	-	-	-	-	-	-	-
	SA Mean	11.6	17.6	17.4	18.2	19.0	18.2	20.7	21.6	22.9
	(±SD)	(14.0)	(16.4)	(14.5)	(16.5)	(17.5)	(16.1)	(18.8)	(19.5)	(18.9)
	Bonferroni	(D)	(C)	(C)	(BC)	(BC)	(BC)	(ABC)	(AB)	(A)
Total Lipids (g)	DRI	-	-	-	-	-	-	-	-	-
	SA Mean	30.6	32.2	32.6	33.7	37.6	38.0	42.2	42.9	53.1
	(±SD)	(19.3)	(21.4)	(21.5)	(22.8)	(28.5)	(24.7)	(28.2)	(30.0)	(36.8)
	Bonferroni	(F)	(F)	(EF)	(DEF)	(CDE)	(BCD)	(BC)	(B)	(A)
Saturated Fat (g)	DRI	-	-	-	-	-	-	-	-	-
	SA Mean	9.3	10.4	9.9	10.3	11.3	11.0	12.3	12.4	15.1
	(±SD)	(8.0)	(8.3)	(7.3)	(8.1)	(10.1)	(8.4)	(9.5)	(10.5)	(11.0)
	Bonferroni	(D)	(CD)	(CD)	(CD)	(BC)	(BCD)	(B)	(B)	(A)
MUFA (g)	DRI	-	-	-	-	-	-	-	-	-
	SA Mean	9.0	10.5	10.8	11.3	12.7	12.6	14.2	14.4	18.1
	(±SD)	(6.6)	(7.3)	(7.7)	(8.6)	(10.6)	(9.2)	(10.9)	(11.1)	(13.9)
	Bonferroni	(F)	(EF)	(DEF)	(CDE)	(BC)	(BCD)	(B)	(B)	(A)
PUFA (g)	DRI	-	-	-	-	-	-	-	-	-
	SA Mean	8.3	8.4	8.9	9.0	10.3	10.9	12.0	12.2	15.3
	(±SD)	(6.1)	(7.4)	(7.8)	(7.3)	(8.8)	(8.7)	(9.7)	(10.1)	(12.5)
	Bonferroni	(E)	(E)	(DE)	(DE)	(CD)	(BC)	(BC)	(B)	(A)
n3 Fatty Acids (g)	AI	0.7	0.7	0.7	0.9	0.9	0.9	0.9	0.9	1.2
	SA Mean	0.3	0.3	0.3	0.3	0.3	0.3	0.4	0.4	0.4
	(±SD)	(0.5)	(0.6)	(0.7)	(0.5)	(0.5)	(0.6)	(0.5)	(0.5)	(0.5)
	Bonferroni	(A)	(A)	(A)	(A)	(A)	(A)	(A)	(A)	(A)
Cholesterol (mg)	DRI	-	-	-	-	-	-	-	-	-
	SA Mean	82.4	109.9	118.6	114.9	121.1	121.8	121.2	135.1	168.7
	(±SD)	(125.7)	(151.6)	(162.3)	(161.3)	(161.2)	(157.9)	(174.6)	(165.0)	(239.7)
	Bonferroni	(C)	(BC)	(B)	(BC)	(B)	(B)	(B)	(AB)	(A)
Trans-fat (g)	DRI	-	-	-	-	-	-	-	-	-
	SA Mean	0.4	0.6	0.6	0.6	0.6	0.5	0.7	0.7	0.8
	(±SD)	(0.6)	(0.8)	(0.7)	(0.8)	(0.9)	(0.8)	(1.0)	(1.1)	(0.9)
	Bonferroni	(C)	(B)	(B)	(B)	(B)	(BC)	(AB)	(AB)	(A)
Total Carbohydrates (g)	EAR	100	100	100	100	100	100	100	100	100
	SA Mean	165.5	179.8	193.9	201.0	218.7	231.6	243.0	236.3	251.9
	(±SD)	(77.6)	(82.0)	(93.1)	(87.3)	(99.8)	(97.1)	(104.5)	(104.5)	(101.8)
	Bonferroni	(G)	(FG)	(EF)	(DE)	(CD)	(BC)	(AB)	(ABC)	(A)
Fibre (g)	EAR Boys	19	19	19	25	25	25	25	25	31
	Girls	19	19	19	25	25	25	25	25	26
	SA Mean	11.6	12.4	13.7	14.3	16.0	16.8	18.1	17.9	17.1
	(±SD)	(8.0)	(7.3)	(8.4)	(8.1)	(9.8)	(9.2)	(12.4)	(12.4)	(8.9)
	Bonferroni	(E)	(DE)	(D)	(CD)	(BC)	(AB)	(A)	(AB)	(AB)
Added Sugar (g)	DRI	-	-	-	-	-	-	-	-	-
	SA Mean	14.7	22.1	24.2	26.2	23.6	25.4	25.8	25.6	26.7
	(±SD)	(16.3)	(20.9)	(24.2)	(31.6)	(25.6)	(24.9)	(22.7)	(24.2)	(26.1)
	Bonferroni	(B)	(A)	(A)	(A)	(A)	(A)	(A)	(A)	(A)

kJ = Kilojoules; EER = Estimated Energy Requirements; PAL = Physical Activity Level; SA = South African; SD = Standard Deviation; kcal = Kilocalories; EAR = Estimated Average Requirements (mass/day); AI = Adequate Intake (mass/day); g = grams; mg = milligrams; PUFA = polyunsaturated fatty acids; MUFA = monounsaturated fatty acids; n3 = omega-3; n6 = omega-6. * Bonferroni multiple comparison: significant differences between mean ± SD values of different age groups depicted with different letters, *p* < 0.05.

**Table 4:** South African child mean ± SD intake of minerals and electrolytes by year of age.

		1 Year	2 Years	3 Years	4 Years	5 Years	6 Years	7 Years	8 Years	9 Years
	*n* =	1018	851	828	752	585	517	360	355	146
Calcium (mg)	AI	500	500	500	800	800	800	800	800	1300
	SA Mean	373.1	322.6	296.9	285.6	305.1	293.1	322.4	304.9	300.2
	(±SD)	(355.0)	(312.3)	(271.5)	(252.6)	(257.2)	(255.5)	(253.8)	(265.7)	(262.1)
	Bonferroni	(A)	(AB)	(B)	(B)	(B)	(B)	(AB)	(B)	(B)
Iron (mg)	EAR Boys	3.0	3.0	3.0	4.1	4.1	4.1	4.1	4.1	5.9
	Girls	3.0	3.0	3.0	4.1	4.1	4.1	4.1	4.1	5.7
	SA Mean	7.6	8.3	9.1	9.7	10.9	11.3	11.7	11.9	12.5
	(±SD)	(4.9)	(4.8)	(5.0)	(4.9)	(6.1)	(5.4)	(5.9)	(6.8)	(5.9)
	Bonferroni	(E)	(DE)	(CD)	(C)	(B)	(B)	(AB)	(AB)	(A)
Magnesium (mg)	EAR	65	65	65	110	110	110	110	110	200
	SA Mean	161.8	175.3	186.7	187.8	208.0	221.2	228.8	220.8	223.3
	(±SD)	(93.5)	(94.4)	(105.6)	(100.6)	(118.0)	(116.6)	(128.3)	(126.5)	(101.8)
	Bonferroni	(D)	(CD)	(BC)	(BC)	(AB)	(A)	(A)	(A)	(A)
Phosphorus (mg)	EAR	380	380	380	405	405	405	405	405	1055
	SA Mean	537.0	573.4	592.0	596.2	641.2	670.2	704.5	701.3	723.6
	(±SD)	(316.1)	(298.5)	(300.9)	(285.8)	(320.3)	(320.9)	(321.95)	(337.7)	(326.2)
	Bonferroni	(D)	(D)	(CD)	(CD)	(BC)	(AB)	(AB)	(AB)	(A)
Potassium (mg)	AI	2000	2000	2000	2000	2300	2300	2300	2300	2500
	SA Mean	1064.7	1106.0	1119.5	1161.8	1255.3	1324.0	1395.5	1367.8	683.2
	(±SD)	(591.7)	(574.8)	(584.0)	(541.8)	(684.6)	(660.6)	(698.9)	(689.2)	(1430.9)
	Bonferroni	(D)	(D)	(D)	(CD)	(BC	(AB)	(A)	(AB)	(A)
Sodium (mg)	EAR	1000	1000	1000	1200	1200	1200	1200	1200	1500
	SA Mean	510.6	769.4	835.6	961.1	1066.3	1110.2	1227.4	1254.2	1693.0
	(±SD)	(496.2)	(613.5)	(657.8)	(732.6)	(825.3)	(825.3)	(896.8)	(843.0)	(1188.2)
	Bonferroni	(F)	(E)	(DE)	(CD)	(C)	(BC)	(B)	(B)	(A)
Zinc (mg)	EAR	2.2	2.2	2.2	4.0	4.0	4.0	4.0	4.0	7.0
	SA Mean	6.4	7.1	7.6	8.0	8.8	9.3	9.6	9.6	10.1
	(±SD)	(3.6)	(3.9)	(3.9)	(4.1)	(4.8)	(5.0)	(5.3)	(5.6)	(4.8)
	Bonferroni	(E)	(DE)	(D)	(CD)	(BC)	(AB)	(AB)	(AB)	(A)
Selenium (mcg)	EAR	17	17	17	23	23	23	23	23	35
	SA Mean	21.1	28.0	29.1	32.3	33.4	33.5	37.2	39.0	51.1
	(±SD)	(20.4)	(29.0)	(29.7)	(31.6)	(33.5)	(30.6)	(32.2)	(33.9)	(41.2)
	Bonferroni	(E)	(D)	(D)	(CD)	(BCD)	(BCD)	(BC)	(B)	(A)

SD = Standard Deviation; SA = South African; EAR = Estimated Average Requirements (mass/day); AI = Adequate Intake (mass/day); g = grams; mg = milligrams; mcg = micrograms. Bonferroni multiple comparison: significant differences between mean ± SD values of different age groups depicted with different letters, *p* < 0.05.

**Table 5:** South African child mean ± SD intake of vitamins by year of age.

		1 Year	2 Years	3 Years	4 Years	5 Years	6 Years	7 Years	8 Years	9 Years
	*n* =	1018	851	828	752	585	517	360	355	146
Vitamin A (mcg)	EAR Boys	210.0	210.0	210.0	275.0	275.0	275.0	275.0	275.0	445.0
	Girls	210.0	210.0	210.0	275.0	275.0	275.0	275.0	275.0	420.0
	SA Mean	598.2	567.68	602.6	639.5	679.3	720.4	762.4	678.1	808.1
	(±SD)	(600.3)	(875.80)	(820.8)	(1072.3)	(996.9)	(1008.7)	(1057.9)	(796.0)	(1111.3)
	Bonferroni	(B)	(B)	(B)	(AB)	(AB)	(AB)	(AB)	(AB)	(A)
Thiamin (mg)	EAR	0.4	0.4	0.4	0.5	0.5	0.5	0.5	0.5	0.7
	SA Mean	1.1	1.11	1.2	1.3	1.4	1.5	1.5	1.5	1.6
	(±SD)	(0.6)	(0.6)	(0.7)	(0.7]	(0.8)	(0.8)	(0.8)	(0.8)	(0.7)
	Bonferroni	(E)	(DE)	(D)	(CD)	(BC)	(AB)	(AB)	(AB)	(A)
Riboflavin (mg)	EAR	0.4	0.4	0.4	0.5	0.5	0.5	0.5	0.5	0.8
	SA Mean	0.8	0.83	0.8	0.9	0.9	0.9	1.0	1.0	1.1
	(±SD)	(0.6)	(0.7)	(0.6)	(0.7)	(0.7)	(0.7)	(0.6)	(0.8)	(0.8)
	Bonferroni	(C)	(BC)	(BC)	(BC)	(BC)	(BC)	(AB)	(AB)	(A)
Niacin (mg)	EAR	5.0	5.0	5.0	6.0	6.0	6.0	6.0	6.0	6.0
	SA Mean	10.7	13.29	14.5	15.6	17.2	18.2	19.7	19.7	21.9
	(±SD)	(6.1)	(7.2)	(6.9)	(7.8)	(8.7)	(8.8)	(9.6)	(9.6)	(10.0)
	Bonferroni	(G)	(F)	(EF)	(DE)	(CD)	(BC)	(B)	(B)	(A)
Vitamin B_6_ (mg)	EAR	0.4	0.4	0.4	0.5	0.5	0.5	0.5	0.5	0.8
	SA Mean	1.5	1.85	2.1	2.4	2.6	2.8	2.9	3.0	3.3
	(±SD)	(0.9)	(1.1)	(1.2)	(1.4)	(1.6)	(1.7)	(1.6)	(1.8)	(1.9)
	Bonferroni	(G)	(F)	(EF)	(DE)	(CD)	(BC)	(B)	(B)	(A)
Folate (mcg)	EAR	120	120	120	160	160	160	160	160	250
	SA Mean	270.1	280.89	309.4	311.4	345.0	382.0	390.6	362.3	391.0
	(±SD)	(184.9)	(188.3)	(221.8)	(206.1)	(232.7)	(242.3)	(266.4)	(244.1)	(248.7)
	Bonferroni	(C)	(C)	(BC)	(BC)	(AB)	(A)	(A)	(A)	(A)
Vitamin B_12_ (mcg)	EAR	0.7	0.7	0.7	1.0	1.0	1.0	1.0	1.0	1.5
	SA Mean	1.8	2.69	2.6	2.9	2.9	3.3	3.2	3.3	3.8
	(±SD)	(3.8)	(9.4)	(6.9)	(11.5)	(9.0)	(9.9)	(9.2)	(8.5)	(9.5)
	Bonferroni	(B)	(AB)	(AB)	(AB)	(AB)	(AB)	(AB)	(AB)	(A)
Vitamin C (mg)	EAR	13.0	13.0	13.0	22.0	22.0	22.0	22.0	22.0	39.0
	SA Mean	43.7	31.89	26.8	27.3	35.3	36.3	52.3	35.0	37.9
	(±SD)	(55.1)	(66.2)	(40.2)	(42.9)	(60.4)	(68.5)	(288.6)	(52.0)	(60.1)
	Bonferroni	(AB)	(B)	(B)	(B)	(AB)	(AB)	(A)	(AB)	(AB)
Vitamin D (mcg)	AI	5.0	5.0	5.0	5.0	5.0	5.0	5.0	5.0	5.0
	SA Mean	2.6	2.08	2.2	2.0	2.3	2.6	2.5	2.8	2.9
	(±SD)	(3.9)	(3.3)	(3.3)	(2.9)	(3.3)	(3.7)	(3.3)	(3.4)	(4.2)
	Bonferroni	(ABC)	(BC)	(ABC)	(C)	(ABC)	(ABC)	(ABC)	(AB)	(A)
Vitamin E (mg)	EAR	5.0	5.0	5.0	6.0	6.0	6.0	6.0	6.0	9.0
	SA Mean	5.3	4.74	4.9	4.8	5.4	5.9	6.5	6.6	8.3
	(±SD)	(4.4)	(4.7)	(4.8)	(4.3)	(4.8)	(5.1)	(5.8)	(6.0)	(7.1)
	Bonferroni	(CD)	(D)	(CD)	(CD)	(CD)	(BC)	(B)	(B)	(A)

SA = South African; SD = Standard Deviation; EAR = Estimated Average Requirements (mass/day); AI = Adequate Intake (mass/day); g = grams; mg = milligrams; mcg = micrograms. Bonferroni multiple comparison: significant differences between mean ± SD values of different age groups depicted with different letters, *p* < 0.05.

**Table 6:** South African child mean ± SD intake of flavonoids and other food parameters by year of age.

		1 Year	2 Years	3 Years	4 Years	5 Years	6 Years	7 Years	8 Years	9 Years
	*n* =	1018	851	828	752	585	517	360	355	146
Flavan-3-ols (mg)	DRI	-	-	-	-	-	-	-	-	-
	SA Mean	59.5	107.4	109.3	111.7	113.2	115.7	116.5	106.4	87.9
	(±SD)	(132.4)	(156.0)	(154.4)	(163.9)	(155.1)	(160.7)	(152.0)	(157.2)	(134.9)
	Bonferroni	(B)	(A)	(A)	(A)	(A)	(A)	(A)	(A)	(AB)
Flavones (mg)	DRI	-	-	-	-	-	-	-	-	-
	SA Mean	0.4	0.6	0.9	0.7	0.9	1.6	2.1	1.4	1.7
	(±SD)	(2.9)	(2.2)	(5.4)	(2.6)	(3.0)	(8.9)	(14.0)	(5.1)	(5.2)
	Bonferroni	(C)	(BC)	(ABC)	(BC)	(BC)	(ABC)	(A)	(ABC)	(AB)
Flavonols (mg)	DRI	-	-	-	-	-	-	-	-	-
	SA Mean	4.0	6.2	6.2	6.3	6.6	6.9	7.8	6.4	6.2
	(±SD)	(6.1)	(6.8)	(6.8)	(6.9)	(6.6)	(7.7)	(7.9)	(7.1)	(6.8)
	Bonferroni	(C)	(B)	(B)	(B)	(AB)	(AB)	(A)	(AB)	(B)
Flavonones (mg)	DRI	-	-	-	-	-	-	-	-	-
	SA Mean	3.8	3.6	3.2	2.8	3.8	3.9	4.4	3.8	3.7
	(±SD)	(14.0)	(13.7)	(13.1)	(12.0)	(13.0)	(14.7)	(15.4)	(15.9)	(13.0)
	Bonferroni	(A)	(A)	(A)	(A)	(A)	(A)	(A)	(A)	(A)
Anthocyanidins (mg)	DRI	-	-	-	-	-	-	-	-	-
	SA Mean	0.5	0.6	1.1	0.8	1.6	1.5	1.2	2.8	0.3
	(±SD)	(4.7)	(5.7)	(8.7)	(9.6)	(18.4)	(13.3)	(8.9)	(15.2)	(1.8)
	Bonferroni	(B)	(AB)	(AB)	(AB)	(AB)	(AB)	(AB)	(A)	(B)
Isoflavones (mg)	DRI	-	-	-	-	-	-	-	-	-
	SA Mean	0.2	0.3	0.4	0.4	0.5	0.5	0.6	0.6	0.9
	(±SD)	(0.8)	(0.5)	(1.3)	(0.7)	(1.5)	(1.1)	(1.5)	(1.5)	(3.1)
	Bonferroni	(E)	(DE)	(BCDE)	(CED)	(BC)	(BCD)	(BC)	(B)	(A)
b-carotene (mg)	DRI	-	-	-	-	-	-	-	-	-
	SA Mean	902.3	882.2	949.7	1039.6	1180.2	1047.9	1287.9	1099.6	963.9
	(±SD)	(2358.6)	(2012.3)	(2019.6)	(2134.2)	(2694.2)	(2154.2)	(2615.7)	(1876.1)	(1705.4)
	Bonferroni	(A)	(A)	(A)	(A)	(A)	(A)	(A)	(A)	[A]
Caffeine (mg)	DRI	-	-	-	-	-	-	-	-	-
	SA Mean	12.5	22.5	24.4	26.9	26.4	30.0	26.8	27.0	28.5
	(±SD)	(27.0)	(32.2)	(33.8)	(36.4)	(34.3)	(45.1)	(34.9)	(36.8)	(39.9)
	Bonferroni	(C)	(B)	(AB)	(AB)	(AB)	(A)	(AB)	(AB)	(AB)
Onion (g)	DRI	-	-	-	-	-	-	-	-	-
	SA Mean	0.7	1.1	0.9	1.2	0.8	0.7	2.0	1.3	3.4
	(±SD)	(7.4)	(6.8)	(6.4)	(9.8)	(7.4)	(6.9)	(15.2)	(10.2)	(14.9)
	Bonferroni	(B)	(B)	(B)	(B)	(B)	(B)	(AB)	(B)	(A)
Tea (g)	DRI	-	-	-	-	-	-	-	-	-
	SA Mean	0.8	1.1	1.1	1.1	1.0	1.1	1.1	1.0	0.9
	(±SD)	(1.4)	(1.3)	(1.3)	(1.3)	(1.2)	(1.3)	(1.20)	(1.2)	(1.1)
	Bonferroni	(B)	(A)	(A)	(A)	(AB)	(A)	(A)	(AB)	(AB)

SD = Standard Deviation; SA = South African; EAR = Estimated Average Requirements (mass/day); AI = Adequate Intake (mass/day); g = grams; mg = milligrams; mcg = micrograms. Bonferroni multiple comparison: significant differences between mean ± SD values of different age groups depicted with different letters, *p* < 0.05.

## Data Availability

The supplementary database is stored on ZivaHub: UCT’s online, institutional data repository that serves as a publishing and access platform to research data and scholarly outputs. DOI:10.25375/uct.17294087.

## Appendix A

**Table A1:** South African child mean ± SD intake of energy and macronutrients by PDIS age groups.

	Age Group	1–2 Years	3–5 Years	6–9 Years	ALL
*n* =		1869	2165	1378	5412
Energy (kJ)	SA Mean	4632.6	5391.1	6381.8	5381.4
	(±SD)	(2047.6)	(2276.9)	(2584.4)	(2381.0)
	Bonferroni	(C)	(B)	(A)	
Energy (kcal)	SA Mean	1108.3	1289.7	1526.7	1287.4
	(±SD)	(489.9)	(544.7)	(618.3)	(569.6)
	Bonferroni	(C)	(B)	(A)	
Total Protein (g)	SA Mean	32.3	39.4	46.2	38.7
	(±SD)	(18.5)	(19.5)	(21.9)	(20.5)
	Bonferroni	(C)	(B)	(A)	
Plant Protein (g)	SA Mean	15.5	19.6	24.0	19.3
	(±SD)	(9.0)	(10.7)	(12.3)	(11.1)
	Bonferroni	(C)	(B)	(A)	
Animal Protein (g)	SA Mean	14.3	18.1	20.2	17.3
	(±SD)	(15.4)	(16.1)	(18.1)	(16.6)
	Bonferroni	(C)	(B)	(A)	
Total Lipids (g)	SA Mean	31.3	34.3	42.0	35.3
	(±SD)	(20.3)	(24.1)	(28.8)	(24.5)
	Bonferroni	(C)	(B)	(A)	
Saturated Fat (g)	SA Mean	9.8	10.4	12.1	10.6
	(±SD)	(8.1)	(8.4)	(9.6)	(8.7)
	Bonferroni	(B)	(B)	(A)	
MUFA (g)	SA Mean	9.7	11.5	14.1	11.5
	(±SD)	(7.0)	(8.9)	(10.8)	(9.0)
	Bonferroni	(C)	(B)	(A)	
PUFA (g)	SA Mean	8.3	9.3	12.0	9.6
	(±SD)	(6.7)	(7.9)	(9.8)	(8.2)
	Bonferroni	(C)	(B)	(A)	
*n*-3 Fatty Acids (mg)	SA Mean	0.3	0.3	0.4	0.3
	(±SD)	(0.5)	(0.6)	(0.5)	(0.5)
	Bonferroni	(B)	(AB)	(A)	
*n*-6 Fatty Acids (mg)	SA Mean	1.2	0.4	0.6	0.7
	(±SD)	(33.1)	(1.7)	(2.0)	(19.5)
	Bonferroni	(A)	(A)	(A)	
Cholesterol (mg)	SA Mean	95.0	118.0	130.0	113.1
	(±SD)	(138.7)	(161.6)	(174.8)	(158.3)
	Bonferroni	(B)	(A)	(A)	
Trans Fat (g)	SA Mean	0.5	0.6	0.7	0.6
	(±SD)	(0.7)	(0.8)	(0.9)	(0.8)
	Bonferroni	(C)	(B)	(A)	
Total Carbohydrates (g)	SA Mean	172.0	203.1	237.9	201.2
	(±SD)	(80.0)	(93.5)	(101.6)	(94.7)
	Bonferroni	(C)	(B)	(A)	
Fibre (g)	SA Mean	12.0	14.5	17.5	14.4
	(±SD)	(7.7)	(8.8)	(10.9)	(9.3)
	Bonferroni	(C)	(B)	(A)	
Added Sugar (g)	SA Mean	18.1	24.7	25.7	22.7
	(±SD)	(18.8)	(27.3)	(24.3)	(24.1)
	Bonferroni	(B)	(A)	(A)	

SD = Standard Deviation; PDIS = Provincial Dietary Intake Study; kJ = Kilojoules; EER = Estimated Energy Requirements; PAL = Physical Activity Level; SA = South African; kCal = Kilocalories; EAR = Estimated Average Requirements (mass/day); AI = Adequate Intake (mass/day); g = grams; mg = milligrams; PUFA = polyunsaturated fatty acids; MUFA = monounsaturated fatty acids; *n*-3 = omega-3; *n*-6 = omega-6. Bonferroni multiple comparison: significant differences between mean ± SD values of different age groups depicted with different letters, *p* < 0.05.

**Table A2:** South African child mean ± SD intake of minerals and electrolytes by PDIS age groups.

Age Group		1–2 Years	3–5 Years	6–9 Years	ALL
*n* =		1869	2165	1378	5412
Calcium (mg)	SA Mean	350.1	295.2	304.5	316.5
	(±SD)	(337.0)	(261.2)	(258.4)	(290.0)
	Bonferroni	(A)	(B)	(AB)	
Iron (mg)	SA Mean	7.9	9.8	11.7	9.6
	(±SD)	(4.9)	(5.3)	(6.0)	[5.6]
	Bonferroni	(C)	(B)	(A)	
Magnesium (mg)	SA Mean	167.9	192.8	223.3	192.0
	(±SD)	(94.1)	(107.8)	(120.8)	(108.9)
	Bonferroni	(C)	(B)	(A)	
Phosphorus (mg)	SA Mean	553.6	606.7	692.9	610.3
	(±SD)	(308.7)	(301.8)	(326.3)	(315.1)
	Bonferroni	(C)	(B)	(A)	
Potassium (mg)	SA Mean	1083.5	1170.9	1365.3	1190.2
	(±SD)	(584.3)	(601.4)	(680.8)	(626.3)
	Bonferroni	(C)	(B)	(A)	
Sodium (mg)	SA Mean	628.5	941.5	1239.7	909.3
	(±SD)	(567.4)	(737.6)	(908.0)	(770.7)
	Bonferroni	(C)	(B)	(A)	
Zinc (mg)	SA Mean	6.7	8.1	9.6	8.0
	(±SD)	(3.8)	(4.3)	(5.2)	(4.5)
	Bonferroni	(C)	(B)	(A)	
Selenium (mcg)	SA Mean	24.3	31.4	37.7	30.5
	(±SD)	(25.0)	(31.4)	(33.5)	(30.4)
	Bonferroni	(C)	(B)	(A)	

SD = Standard Deviation; PDIS = Provincial Dietary Intake Study; SA = South African; EAR = Estimated Average Requirements (mass/day); AI = Adequate Intake (mass/day); g = grams; mg = milligrams; mcg: micrograms. Bonferroni multiple comparison: significant differences between mean ± SD values of different age groups depicted with different letters, *p* < 0.05.

**Table A3:** South African child mean ± SD intake of vitamins by PDIS age groups.

	Age Group	1–2 Years	3–5 Years	6–9 Years	ALL
*n* =		1869	2165	1378	5412
Vitamin A (mcg)	SA Mean	584.3	636.1	729.8	642.1
	(±SD)	(738.5)	(962.1)	(983.7)	(898.8)
	Bonferroni	(B)	(B)	(A)	
Thiamin (mg)	SA Mean	1.1	1.3	1.5	1.3
	(±SD)	(0.6)	(0.7)	(0.8)	(0.7)
	Bonferroni	(C)	(B)	(A)	
Riboflavin (mg)	SA Mean	0.8	0.9	1.0	0.9
	(±SD)	(0.7)	(0.7)	(0.7)	(0.7)
	Bonferroni	(C)	(B)	(A)	
Niacin (mg)	SA Mean	11.9	15.6	19.3	15.3
	(±SD)	(6.8)	(7.8)	(9.4)	(8.4)
	Bonferroni	(C)	(B)	(A)	
Vitamin B_6_ (mg)	SA Mean	1.6	2.3	3.0	2.2
	(±SD)	(1.0)	(1.4)	(1.7)	(1.5)
	Bonferroni	(C)	(B)	(A)	
Folate (mcg)	SA Mean	275.0	319.7	380.1	319.6
	(±SD)	(186.5)	(220.0)	(249.9)	(221.1)
	Bonferroni	(C)	(B)	(A)	
Vitamin B_12_ (mcg)	SA Mean	2.2	2.8	3.3	2.7
	(±SD)	(7.0)	(9.3)	(9.3)	(8.6)
	Bonferroni	(B)	(AB)	(A)	
Vitamin C (mg)	SA Mean	38.3	29.3	40.3	35.2
	(±SD)	(60.7)	(47.5)	(156.8)	(92.0)
	Bonferroni	(A)	(B)	(A)	
Vitamin D (mcg)	SA Mean	2.4	2.2	2.7	2.4
	(±SD)	(3.6)	(3.2)	(3.6)	(3.5)
	Bonferroni	(B)	(AB)	(A)	
Vitamin E (mg)	SA Mean	5.1	5.0	6.5	5.4
	(±SD)	(4.5)	(4.6)	(5.8)	(5.0)
	Bonferroni	(B)	(B)	(A)	

SD = Standard Deviation; PDIS = Provincial Dietary Intake Study; RE = Retinol Equivalents; SA = South African; EAR = Estimated Average Requirements (mass/day); AI = Adequate Intake (mass/day); g = grams; mg = milligrams; mg = micrograms; mcg = micrograms. Bonferroni multiple comparison: significant differences between mean ± SD values of different age groups depicted with different letters, *p* < 0.05.

**Table A4:** South African child mean ± SD intake of flavonoids and other food parameters by PDIS age groups.

	Age Group	1–2 Years	3–5 Years	6–9 Years	ALL
*n* =		1869	2165	1378	5412
Flavan-3-ols (mg)	SA Mean	81.3	111.2	110.6	100.7
	(±SD)	(145.5)	(157.9)	(155.1)	(153.6)
	Bonferroni	(B)	(A)	(A)	
Flavones (mg)	SA Mean	0.5	0.8	1.7	0.9
	(±SD)	(2.6)	(4.0)	(9.5)	(5.7)
	Bonferroni	(B)	(B)	(A)	
Flavonols (mg)	SA Mean	5.0	6.3	7.0	6.0
	(±SD)	(6.5)	(6.8)	(7.5)	(6.9)
	Bonferroni	(C)	(B)	(A)	
Flavonones (mg)	SA Mean	3.7	3.2	4.0	3.6
	(±SD)	(13.9)	(12.7)	(15.0)	(13.7)
	Bonferroni	(A)	(A)	(A)	
Anthocyanidins (mg)	SA Mean	0.5	1.1	1.6	1.1
	(±SD)	(5.2)	(12.3)	(12.2)	(10.4)
	Bonferroni	(B)	(AB)	(A)	
Isoflavones (mg)	SA Mean	0.2	0.4	0.6	0.4
	(±SD)	(0.7)	(1.2)	(1.6)	(1.2)
	Bonferroni	(C)	(B)	(A)	
b-carotene (mg)	SA Mean	893.1	1043.2	1115.0	1009.7
	(±SD)	(2207.1)	(2259.8)	(2178.5)	(2222.5)
	Bonferroni	(B)	(AB)	(A)	
Caffeine (mg)	SA Mean	17.0	25.8	28.2	23.4
	(±SD)	(29.9)	(34.9)	(40.0)	(35.0)
	Bonferroni	(B)	(A)	(A)	
Onion (g)	SA Mean	0.9	1.0	1.5	1.1
	(±SD)	(7.1)	(8.0)	(11.3)	(8.7)
	Bonferroni	(A)	(A)	(A)	
Tea (g)	SA Mean	0.9	1.1	1.0	1.0
	(±SD)	(1.4)	(1.3)	(1.2)	(1.3)
	Bonferroni	(B)	(A)	(A)	

SD = Standard Deviation; PDIS = Provincial Dietary Intake Study; SA = South African; EAR = Estimated Average Requirements (mass/day); g = grams; mg = milligrams; mcg = micrograms. Bonferroni multiple comparison: significant differences between mean ± SD values of different age groups depicted with different letters, *p* < 0.05.
